# Eye-gaze information input based on pupillary response to visual stimulus with luminance modulation

**DOI:** 10.1371/journal.pone.0226991

**Published:** 2020-01-09

**Authors:** Yumiko Muto, Hideka Miyoshi, Hirohiko Kaneko

**Affiliations:** 1 Dept. of Information and Communications Engineering, Tokyo institute of Technology, Yokohama, Kanagawa, Japan; 2 Brain Science Institute, Tamagawa university, Tokyo, Japan; Preeminent Medical Phonics Education & Research Center, Hamamatsu University School of Medicine, JAPAN

## Abstract

This study develops an information-input interface in which a visual stimulus targeted by a user’s eye gaze is identified based on the pupillary light reflex to periodic luminance modulations of the object. Experiment 1 examines how pupil size changes in response to periodic luminance modulation of visual stimuli, and the results are used to develop an algorithm for information input. Experiment 2a examines the effectiveness of interfaces with two objects. The results demonstrate that 98% accurate identification of the gaze targeted object is possible if the luminance modulation frequencies of two objects differ by at least 0.12 Hz. Experiment 2b examines the accuracy of a gaze directed information input method based on a keyboard configuration with twelve responses. The results reveal that keyboard input is possible with an average accuracy of 85% for luminance modulation frequencies from 0.75 to 2.75 Hz. The proposed pupillometry based information-input interface offers several advantages, such as low burden on users, minimal invasiveness, no need for training or experience, high theoretical validity, and no need for calibration. Thus, the pupillometry method presented herein has advantages for practical use without requiring the eye’s position to be calibrated. Additionally, this method has a potential for the design of interfaces that allow patients with severely limited motor function to communicate with others.

## Introduction

To regulate the amount of light entering the eye, the pupil diameter changes as a function of the brightness of the object being viewed [1] [2] [3] [4], and the characteristics of this pupillary light reflex (PLR) have been explored in many studies [e.g., [5] [6] [7] [8]]. The present study develops an information-input interface in which a visual object targeted by the user’s eye gaze is detected based on the pupillary light reflex to periodic luminance modulations of the object. In addition to conventional information-input devices such as the keyboard and mouse, several methods have been proposed that use biological signals such as eye-gaze position and brain waves [see, e.g., [9] [10]]. Unfortunately, the signals produced by such methods have some disadvantages for the intended purpose: The brain-wave techniques generally require a contact sensor, which is extremely expensive and complicated signal analysis to extract the desired information. Conventional eye-gaze input methods require not only user training but also calibration to relate the signal from the device to eye position. In addition, even if individual adjustments are made beforehand, measurements of eye position are not possible in some cases. In contrast, pupillometry offers various advantages for information input. To begin, it requires only a relatively inexpensive noncontact sensor. In addition, pupillary response to light is sufficiently stable and not only requires no calibration to relate the signal to the change in light intensity, but also requires no user training to improve signal stability. Accordingly, pupillometry is a promising technique for information input, especially for patients with extremely limited motor functions, such as those suffering from the locked-in syndrome.

To communicate intentions and as an information-input interface, particularly among the patients with extremely limited motor function, a method has been proposed that uses the pupillary response to a cognitive load, such as two-digit mental arithmetic [11]. This method is used mainly for patients with locked-in syndrome. The study posed yes-no questions and obtained approximately 3.6 selections per minute with 90% accuracy for healthy participants and approximately 2.7 selections per minute with 70% accuracy for patients with severe motor disabilities. However, this accuracy and response frequency are insufficient for practical use, and the technique subjects the patients to an excessive cognitive load. Recently, Mathôt et al. [12] proposed an information-input method to estimate the position of covert visual attention foci that employed pupillometry and that uses visual stimuli consisting of two circles of black and white that flash alternately. Participants selected a letter by looking at it covertly. The letters were shown within circles with oscillating brightness. Small changes in pupil size reflected changes in the luminance of the attended stimulus [13], which enabled the stimulus selected by the participant to be identified in real time independently of eye movement. This algorithm was based on the finding that PLR could be modulated via covert attention [14] [15] and used as a communication method by patients with complete loss of motor control. However, multiple numbers and characters must be displayed simultaneously if it is to be used for actual human–computer interaction (HCI) applications simultaneously. Fundamentally, this method is based on two response options; for example, selecting one character out of eight requires repeating three times the process of selection between two patterns, which requires an excessively long time for information input, making for an inefficient information-input technique. For use in real-life situations, multiple numbers and characters must be displayed simultaneously.

Herein, we propose a method to determine gaze direction based on the frequency of the PLR response to periodic luminance modulations of various visual stimuli. The visual stimulus consists of circles whose luminance is modulated at various frequencies and that demark pieces of input information (numerals or characters). Specifically, the user may be presented with multiple pieces of input information; for example, the numerals 0 to 9. Each numeral is demarked by a circle whose luminance is modulated at a unique frequency (i.e., a frequency that differs from that of the circles around the other nine numerals). As the observer gazes at a circle, the frequency of pupillary oscillation is measured, which allows the item with the same luminance-modulation frequency to be identified as the target of the gaze. This approach determines a unique target of the gaze based only on the pupil data and without directly measuring eye gaze. It can be used with multiple simultaneous stimuli that correspond to various numbers and characters. Previous research used square- or sine-wave-modulated luminance to explore the pupillary response to visual stimuli and clarify its basic characteristics in order to understand the mechanisms and construct a PLR model [see, e.g., [16] [17] [18] [19] [20]]. However, it remains unclear how the pupil responds to visual stimuli consisting of multiple circles whose luminance is modulated at different frequencies. In addition, no reports exist of the PLR applied to an information-input interface based on items whose luminance is modulated at different frequencies.

**Fig 1:**
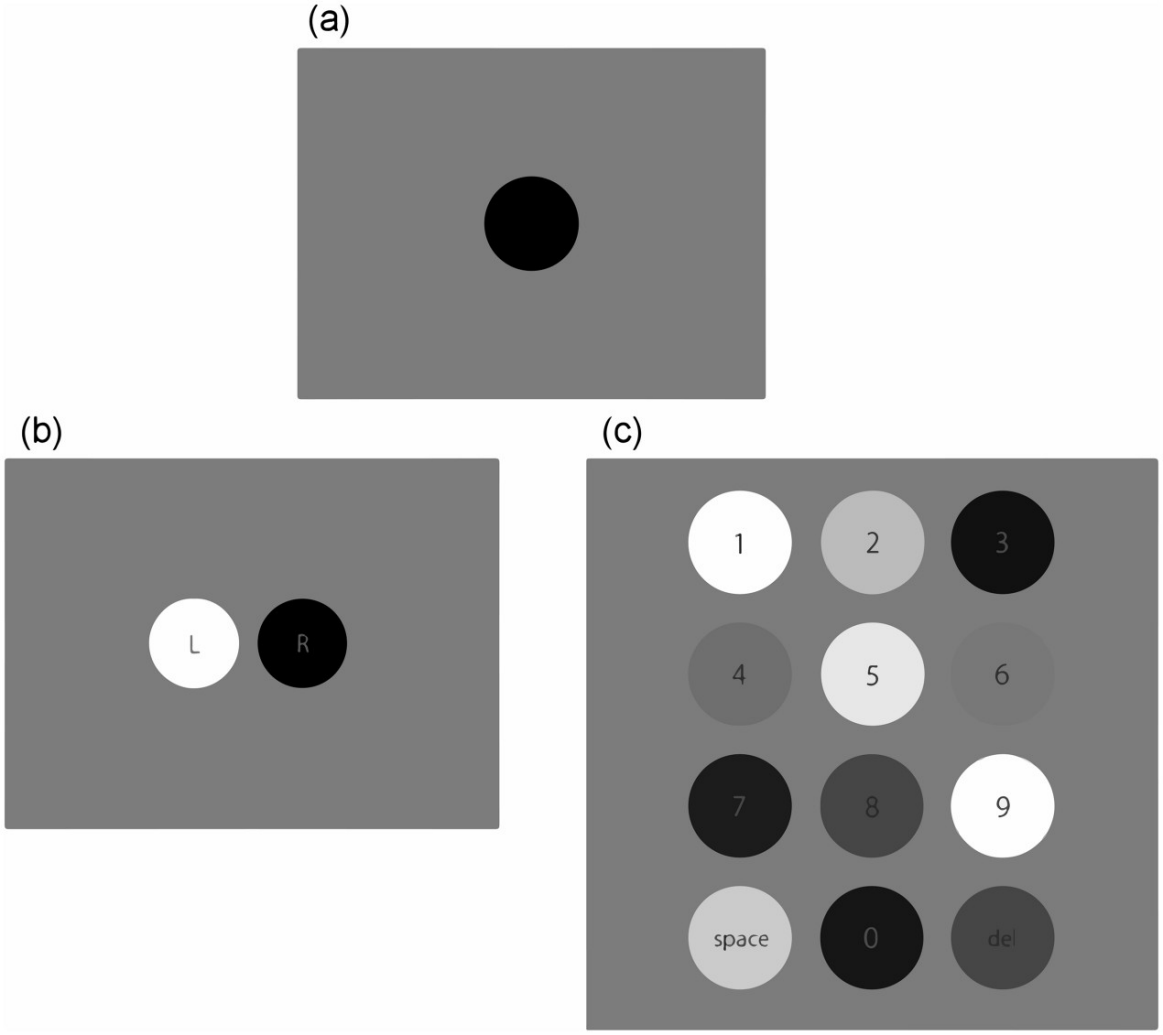
Visual stimuli for (a) experiment 1, (b) experiment 2a, and (c) experiment 2b.

This study consists of two parts: First, we investigate the basic characteristics of pupillary response to a periodic luminance modulation (Experiment 1). Second, we propose an algorithm for a communication system that uses pupillometry and show its effectiveness when applied to an information-input interface in which multiple visual items are presented simultaneously (Experiment 2). As shown in Fig 1, experiment 2a proposes an interface with two response options (yes or no) and experiment 2b proposes an interface in the form of a numeric keypad with twelve response options. Experiment 1 presents a single circular visual stimulus in the center of a screen and modulates its luminance for each trial at one of the following frequencies: 0.25, 0.5, 0.75, 1.0, 1.25, 1.5, 1.75, 2.0, 2.25, 2.5, 2.75 or 3.0 Hz (Fig 1a). The time-domain pupil-diameter data are analyzed by using a discrete Fourier transform (DFT) to quantify the characteristics of the pupil response to the luminance-modulation frequency. This allows us to identify a suitable range of luminance-modulation frequency and to propose an algorithm to estimate the luminance-modulation frequency of the gaze-targeted item based only on the pupil data gathered while the patient gazes at the input interface. Experiment 2 applies the algorithm developed in experiment 1. Experiment 2a allows us to evaluate the effectiveness of an input interface that offers two response options (yes or no) and experiment 2b does the same but for an interface with 12 response options (numerals or characters). Experiment 2a presents two items on a screen (Fig 1b) and the circle gazed at by the observer is identified by comparing the measured frequency of pupil-size oscillation with the luminance-modulation frequency of the various items in the interface. Experiment 2b presents twelve items (characters or numbers) on a screen (Fig 1c) and uses the same approach to identify the gaze-targeted item. Herein we evaluate the accuracy of this input identification method. Herein, participants were instructed to pay attention to a visual stimulus while looking at it. This is referred to as overt attention. Previous studies [11] [12] focused on covert visual attention and intentionally excluded the need for motor (eye) movement. In contrast, our proposed system was designed to use overt attention as a source of new input data.

## Experiment 1: Basic characteristics of pupillary response to periodic luminance modulation

### Method

#### 1. Participants

Ten observers participated in experiment 1, all of which were students and staff (nine males and one female, aged 23 to 29) affiliated with the Tokyo Institute of Technology. All had either normal or corrected to normal vision. The experiment was conducted with the approval of the Ethics Committee of the Tokyo Institute of Technology. Written informed consent was obtained from each participant.

#### 2. Apparatus

Participants were seated in a chair in a dark room in front of a CRT monitor positioned at a viewing distance of 50 cm (Sony Corporation, GDM-F400, 1280 × 1024 pixels, 19 inch). A head-chin rest was used to fix the head position of each participant. Pupil size and eye position of the left eye were recorded with an eye-movement measurement system consisting of infrared illumination and a CCD camera (iRecHS2 system, [21]) with a data-sampling frequency of 333 Hz.

#### 3. Visual stimuli

The visual stimulus used in the experiment consisted of a 3°diameter circle on the gray background (16.4 cd/*m*^2^) of the screen. The luminance of the circle (*y*_*RGB*_) was modulated as follows:

$$\begin{array}{c} y_{RGB}=Asin\left(2\pi ft\right)+127, \end{array}$$ (1)

where *f* is the luminance-modulation frequency [Hz] of the circle and A is the amplitude of the luminance modulation and is fixed at 128, which is half of the maximum RGB value. The visual stimuli were generated and controlled by using MATLAB (MathWorks, Inc.) and a function library (Psychtoolbox-3) installed on a Mac Book Air running OS X Yosemite (Apple Inc.). The gamma value of the CRT monitor was determined to be 2.8 based on measurements with a luminance meter (CS-100A Luminance and Color Mete, KONICA MINOLTA, INC.) and the gamma correction was applied to linear RGB values. The darkest stimulus was 0.99 *cd*/*m*^2^ and the brightest was 99.4 *cd*/*m*^2^.

#### 4. Procedure

The circle was presented in the center of the screen (Fig 1a). The RGB values of this circle were modulated by a sine function to create a grayscale luminance modulation [see Eq (1)]. Twelve luminance-modulation frequencies were tested: 0.25, 0.5, 0.75, 1.0, 1.25, 1.5, 1.75, 2.0, 2.25, 2.5, 2.75, and 3.0 Hz. The frequencies were selected based on the study [22], which shows that a temporal series of luminance stimuli could be synchronized with modulations in pupil size at frequencies up to 3.15 Hz in participants aged from 20 to 30 years. Each trial began by a signal from the participant, and each stimulus was presented for 10 s. Each session consisted of 12 trials, each with a different frequency, and with the 12 frequencies presented in random order. Two sessions were conducted for each participant and no practice trials were held prior to the experiment. Participants started each trial by pressing a start button after closing their eyes briefly and resting for what they deemed sufficient time between each trial. Participants were also instructed to pay attention to the visual stimulus while looking at it.

#### 5. Analysis

The initial second of pupil data was excluded from the analysis because pupil size is known to be influenced by the onset of visual stimulus [6]. The data were then smoothed by applying a moving-average filter with the duration of 120 ms, that is each mean is calculated over a sliding window of length 40 data. After this preprocessing, the frequency characteristics of the data were explored by applying a DFT [23]. Specifically, the frequency with the highest power was determined based on the periodogram power spectral density (PSD), which was estimated from the frequency components obtained from the DFT of the measured and normalized time-domain data of pupil size. The frequency steps in the DFT were approximately 0.01 Hz [24]. A periodogram is a nonparametric method for estimating PSD and is defined below. Comparative reduction of noise components is an advantage of this technique and involves squaring the amplitude of the DFT of a sample and scaling it for comparison with other methods in which a simple spectrum is calculated. Specifically, this is done as follows:

$$\begin{array}{c} P_{xx}\left(f\right)=\frac{1}{LF_{s}}{\left|\sum_{n=0}^{L-1}x_{L}\left(n\right)e^{-j2\pi fn/F_{s}}\right|}^{2}, \end{array}$$ (2)

where *F*_*s*_ is the sampling frequency and *P*_*xx*_(*f*) is the PSD of a signal *x*_*L*_(*n*) with length *L*. All calculations were done offline using the Matlab signal processing toolbox (MathWorks, Inc.). There are two more reasons why we applied the DFT analysis: First, the use of visual stimuli in which the luminance was sinusoidally modulated led us to expect a similar sinusoidal oscillation of the pupil diameter. If the DFT reveals a strong frequency component in the sinusoidally modulated data, this frequency could serve as an appropriate index of synchronization. Second, given two or more visual items with different luminance-modulation frequencies (Experiment 2), the influence of items not targeted by the gazed of the patient may be quantified by comparing the PSD of the luminance-modulation frequencies of these items.

### Results and discussion

#### 1. Frequency analysis of pupillary response

**Fig 2:**
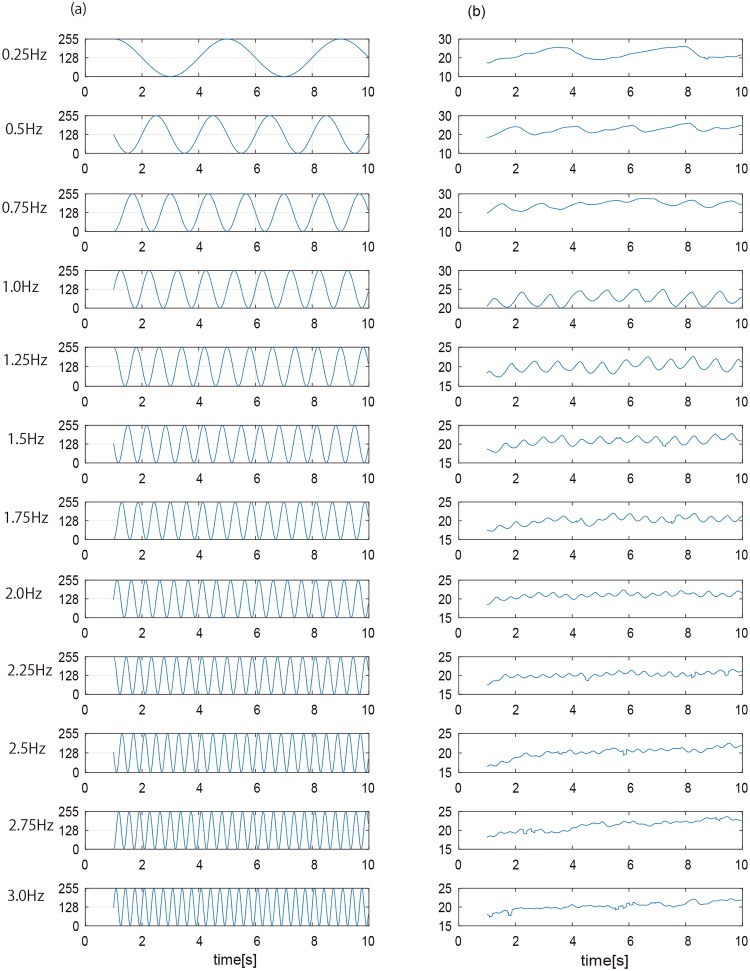
Experiment 1: (a) luminance-modulated visual stimuli and (b) change in pupil size [pix] as a function of time. The initial second of data is excluded from the analysis. See text for details.

**Fig 3:**
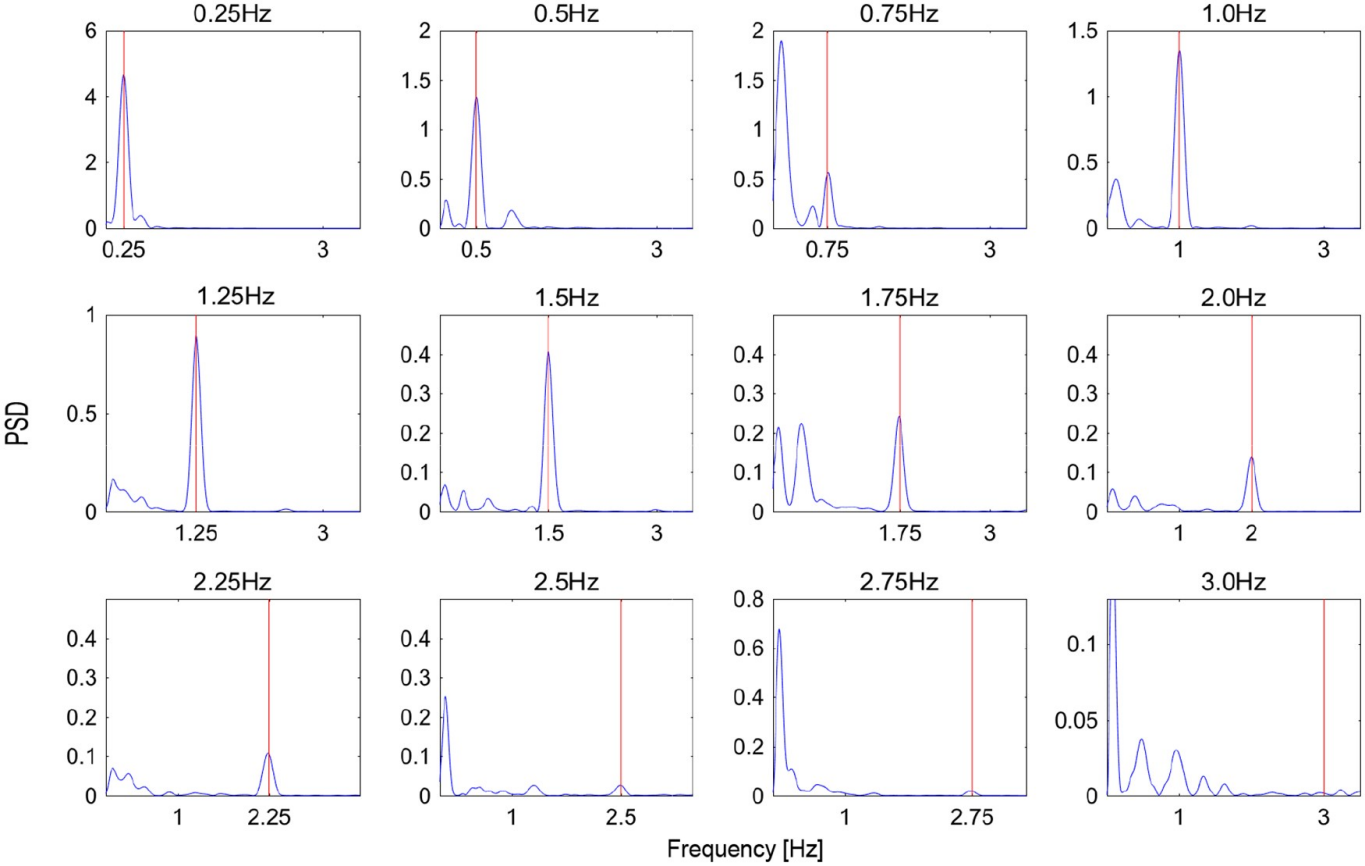
Experiment 1: Examples of DFT of time-domain pupil-size data; the results are for a single participant. The red line shows the luminance-modulation frequency of the gaze-targeted visual stimulus.

In experiment 1, we investigated the time domain data of pupil response to an isolated visual stimulus presented at the center of a screen and with one of 12 different luminance-modulation frequencies. Fig 2 shows examples for one participant of the measured time-domain data for pupil diameter (right column) in response to the 12 luminance-modulation frequencies (left column). Fig 3 shows the PSD obtained as a function of frequency by applying a DFT to the data of Fig 2. The red lines indicate the luminance-modulation frequency for the given visual stimulus.

**Fig 4:**
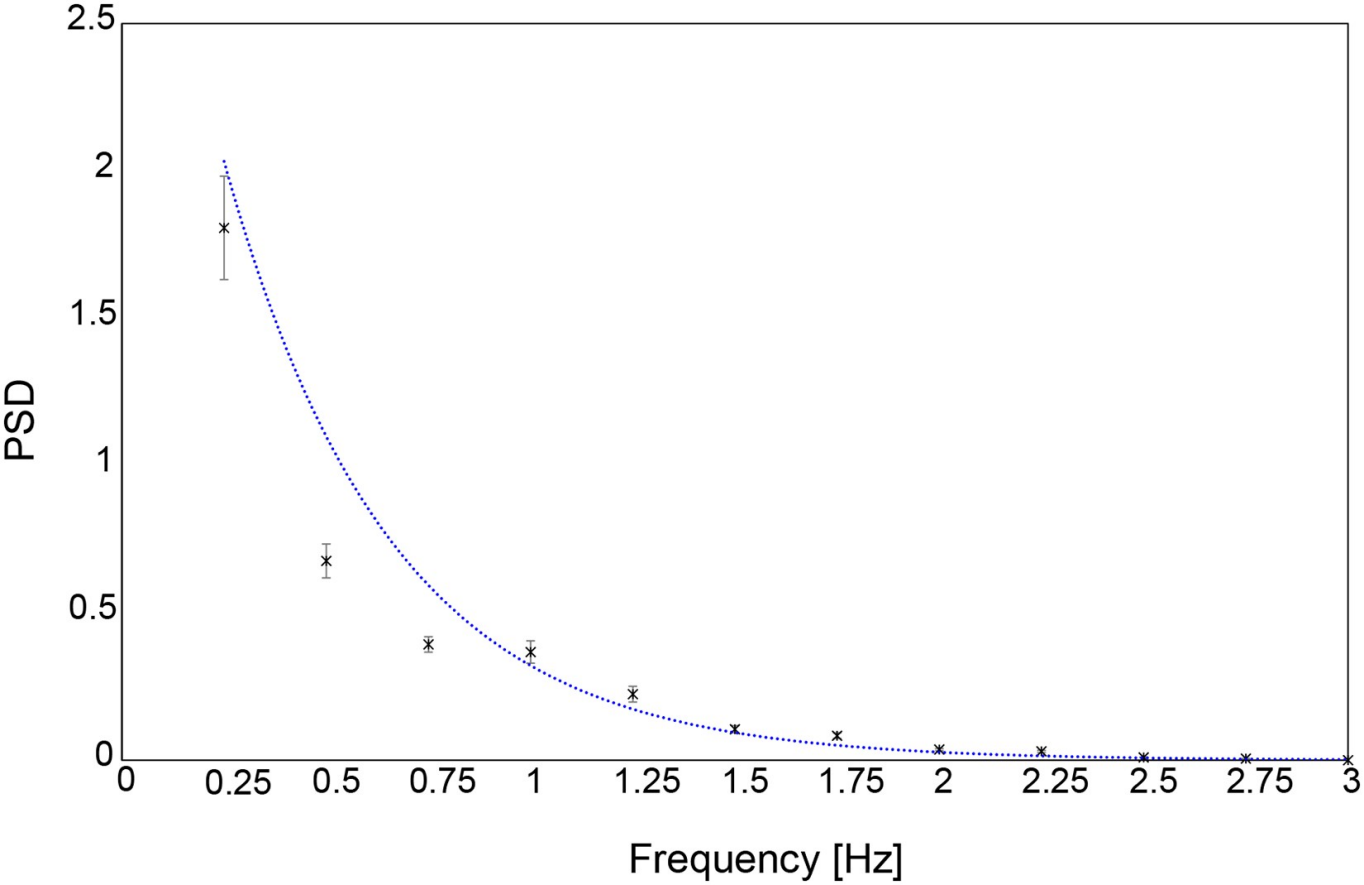
Experiment 1: Average PSD of subjects as a function of luminance-modulation frequency of gaze-targeted visual stimulus.

These results show that, for all luminance-modulation frequencies *f*, the PSD peak, which indicates the main frequency at which the pupil size is modulated, is approximately the same as the luminance-modulation frequency of the gaze-targeted visual stimulus. In other words, the modulation of pupil size is synchronized with the luminance modulation of the visual stimulus, as expected. However, the magnitude of the PSD peak depends on the luminance-modulation frequency of the visual stimulus. Therefore, to quantify the change in PSD frequency, we averaged the PSD peak over all the participants for each luminance-modulation frequency (Fig 4). The results show that the PSD peak decreases with increasing luminance-modulation frequency. A nonlinear regression analysis shows that this relationship may be approximated with high explanatory power by the exponential function

$$\begin{array}{c} f(x)=a\exp(bx) \end{array}$$ (3)

Fitting Eq (3) to the data gives a = 3.545, b = −2.844 (*R*^2^ = 0.97, root mean square error = 0.09). This decrease in PSD peak with increasing frequency is attributed to the fundamental characteristics of the pupil response. Numerous previous studies have shown that the amplitude of pupil-size modulation depends on the luminance modulation frequency of the visual stimulus. The amplitude of pupil-size modulation is small in response to rapid luminance modulations[see, e.g., [6]]. Because the PSD analysis applied herein is based on the amplitude of pupil-size modulations [*Eq*(2)], the exponential decrease in Fig 4 is attributed to the differences in PSD amplitude at each frequency. Therefore, a meaningful comparison of PSD magnitudes in response to luminance-modulation frequencies would require a correction for the fundamental difference in the pupil response amplitude.

#### 2. Estimation of oscillation frequency f of visual stimulus

This study estimates the luminance-modulation frequency *f* of the visual stimulus from the measured oscillations in pupil size. First, for each trial, we calculated the PSD for each of the 12 frequencies (*f* = 0.25, 0.5, 0.75, 1.0, 1.25, 1.5, 1.75, 2.0, 2.25, 2.5, 2.75, or 3.0 Hz). Next, the PSD for each frequency was corrected by multiplying by the weight

$$\begin{array}{c} \omega =\frac{1}{f(x)}=\frac{1}{a}\exp\left(-bx\right), \end{array}$$ (4)

which is the reciprocal of Eq (3), because the PSD varies exponentially with modulation frequency, as shown in Fig 4. We determine the parameters *a* and *b* in the exponential function *f*(*x*) [Eq (3)] from a nonlinear regression analysis of the data (see Fig 4). After this correction, the PSD results for the 12 frequencies are compared to estimate the frequency *f*′ that produces the largest PSD (*PSD*_*max*_). If the estimated frequency *f*′ corresponds to the luminance-modulation frequency *f* of the gaze-targeted visual stimulus, then the estimate of *f*′ is considered successful. Table 1 lists the estimation accuracy averaged over the 10 participants for each frequency. An accuracy of 90% or greater is obtained at all frequencies except for 0.5 and 3.0 Hz, which have accuracies of 80% and 30%, respectively. These results reveal that the proposed method allows us to estimate the gaze point with high accuracy (90%) if the luminance-modulated frequency of the visual stimulus is between 0.75 and 2.75 Hz. Table 2 is the estimation confusion matrix. The first column shows the frequency of the estimated stimulus (Hz), whereas the actual frequency is shown in a horizontal row. Estimation error was highest at 3 Hz, which indicated that 3 Hz was not suitable for application in the system. An upper limit of 2.75 Hz was more appropriate.

**Table 1:** Experiment 1: Accuracy for each frequency.

Frequency[Hz]	0.25	0.5	0.75	1.0	1.25	1.5	1.75	2.0	2.25	2.5	2.75	3.0
Accuracy[%]	90	80	90	90	90	90	100	90	100	100	100	30

**Table 2:** Experiment 1: Confusion matrix.

		Estimated frequency [Hz]											
		0.25	0.5	0.75	1.0	1.25	1.5	1.75	2.0	2.25	2.5	2.75	3.0
Actual frequency [Hz]	0.25	**9**	1	0	0	0	0	0	0	0	0	0	0
	0.5	0	**8**	0	0	0	1	0	0	0	0	0	1
	0.75	0	0	**9**	0	0	0	0	0	0	0	0	1
	1	0	0	0	**9**	0	0	0	0	0	0	0	1
	1.25	0	0	0	0	**9**	0	0	0	0	0	0	1
	1.5	0	0	0	0	0	**9**	0	0	0	0	0	1
	1.75	0	0	0	0	0	0	**10**	0	0	0	0	0
	2	0	0	0	0	0	0	0	**9**	0	0	0	1
	2.25	0	0	0	0	0	0	0	0	**10**	0	0	0
	2.5	0	0	0	0	0	0	0	0	0	**10**	0	0
	2.75	0	0	0	0	0	0	0	0	0	0	**10**	0
	3	3	0	0	1	0	0	0	0	1	2	0	**3**

## Experiment 2: Information-input interface and its evaluation

### Method

#### 1. Participants

Experiment 2a used ten male participants aged 22 to 29 years and experiment 2b used eight participants (five males and three females) aged 21 to 28 years, all of whom were students or staff affiliated with the Tokyo Institute of Technology. All participants had either normal or corrected to normal vision. The two experiments were conducted on different dates and times. One participant participated in both experiments. The experiments were approved by the Ethics Committee of the Tokyo Institute of Technology. A written informed consent was obtained from each study participant.

#### 2. Apparatus and analysis

The apparatus for experiment 2 and its analysis are the same as for experiment 1.

#### 3. Procedure

1. **Experiment 2a: Communication interface with two response options**
  This interface is intended to be used for a communication device that accepts only two answers, such as yes or no. Two circles separated by a 1°viewing angle appear side by side on the screen (Fig 1b) and serve as visual stimuli. The left circle is modulated one of the following eight luminance-modulation frequencies: *f* = 0.75, 1, 1.25, 1.5, 1.75, 2, 2.25, and 2.5 Hz. The luminance-modulation frequency of the right visual stimulus was 0.06, 0.12, or 0.25 Hz higher than that of the left stimulus. For example, when the left circle was modulated at 0.75 Hz, the right circle was modulated at 0.81, 0.87, or 1.0 Hz. In a given experimental session, each combination of stimulus frequencies was presented once, so 3× 8 = 24 trials were conducted in total. The luminance-modulation frequencies of the left and right circles differed by 0.06, 0.12, or 0.25 Hz to determine the smallest possible frequency difference Δ*f* for which the gaze position of the pupil could be differentiated. We assumed that this combination of frequencies could be used to examine how the luminance modulation of the adjacent visual object affects the results. The participants were instructed to direct their gaze at the left or right circle. We refer to the condition under which eye gaze was directed at the right (left) circle as the R (L) condition. Each participant took part in two sessions so that the experiment comprised a total of 48 trials. All of the combinations of stimuli and instructions were presented in random order. Each session was divided into two sets of 12 trials, with each set of trials conducted separately. In each trial, first, a fixation point was presented at the center of a gray background. After participants pressed the start button to begin the trial, an “R” or “L” was displayed, instructing the participants to gaze at either the right or left circle. The luminance of the circles was then modulated for 8 s while the participants directed their gaze at the designated circle. After closing their eyes to rest as needed, the participants pressed the start button for the next trial. No training was done in advance of the experiments.
2. **Experiment 2b: Information-input interface with 12 options**
  This interface is intended to be used as an input device (e.g., a numeral or character keyboard) with a small number of keys. Twelve circles were presented simultaneously on the screen (see Fig 1c), with a 1°viewing-angle separation between the edges of adjacent circles. The luminance-modulation frequencies of the circles were, from left to right, 0.58, 0.70, 0.82 for the top row, 0.94, 1.06, 1.18 for the second row, 1.30, 1.42, 1.54 for the third row, and 1.66, 1.78, 1.90 Hz for the bottom row. These 12 frequencies were separated by 0.12 Hz. At the center of each circle appeared a numeral from 0 through 9, the word “SPACE,” and the symbols “<” or “−” with a viewing-angle size of 0.5°and a fixed luminance of 16.4 *cd*/*m*^2^. The participants started each trial by pressing the start button. First, at the center of the gray screen appeared for 2 s a numeral or character, which instructed the participant to gaze at the corresponding circle during the following trial. The 12 stimuli (Fig 1c) were then presented for 8 s each, during which time the participants gazed at the predesignated circle. Prior to this experiment, each participant took part in a practice session.

#### 4. Results and discussion

**Fig 5:**
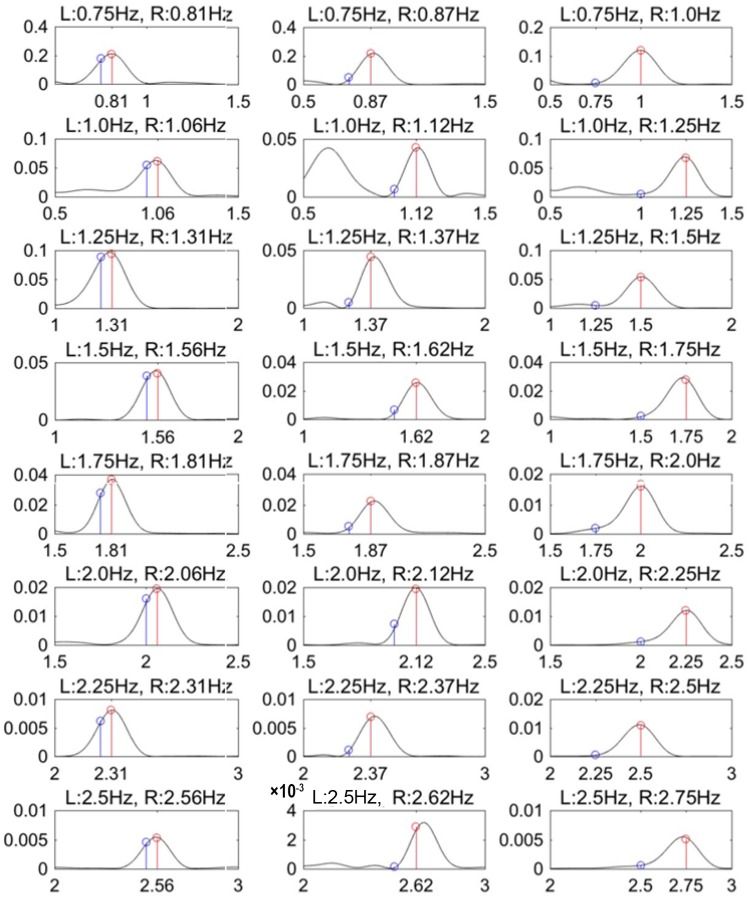
Experiment 2a: Example of DFT of time-domain pupil modulation results for one participant and for each frequency condition in which eye gaze was directed at the right circle.

**Fig 6:**
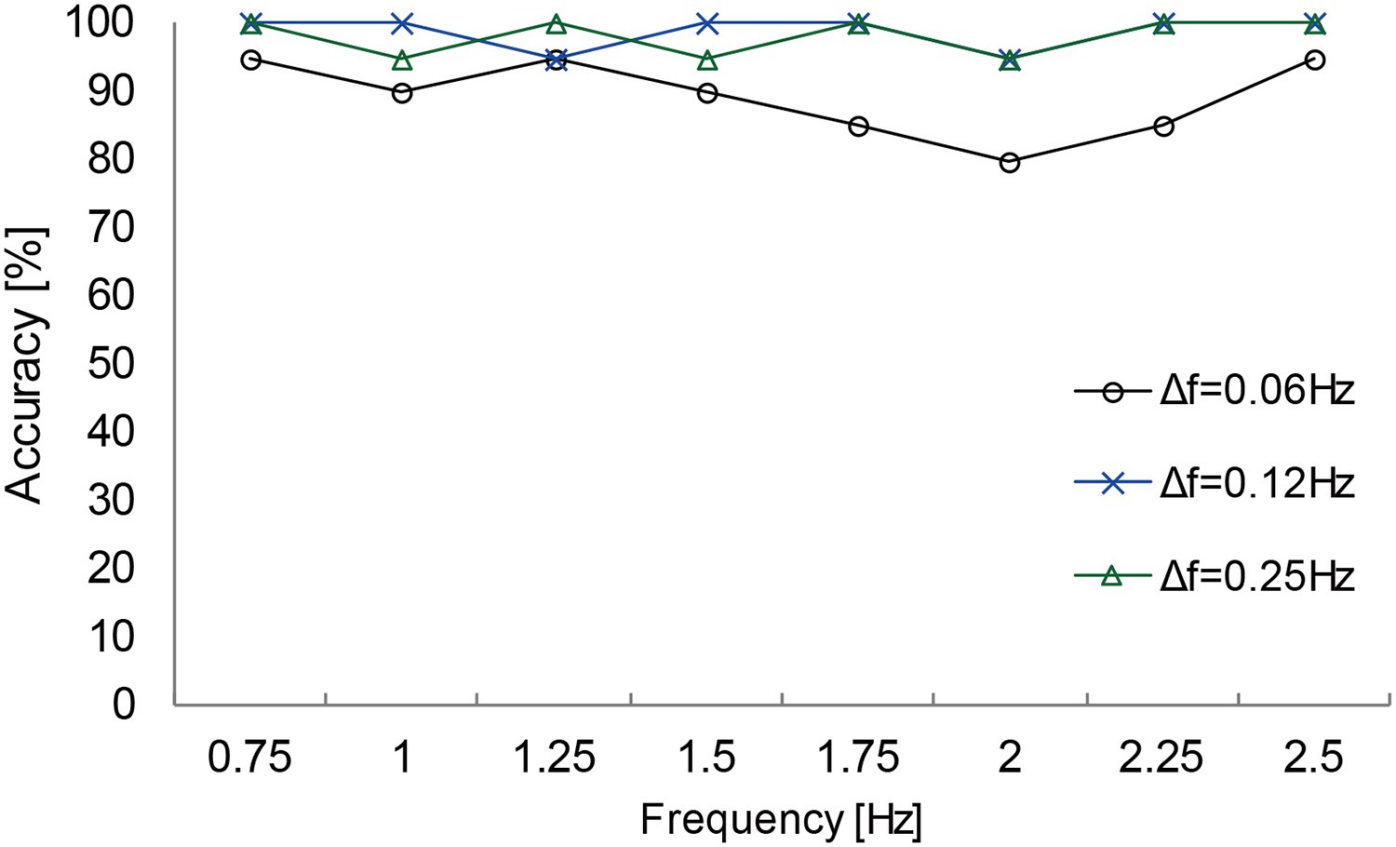
Average identification accuracy as a function of luminance-modulation frequency.

**Fig 7:**
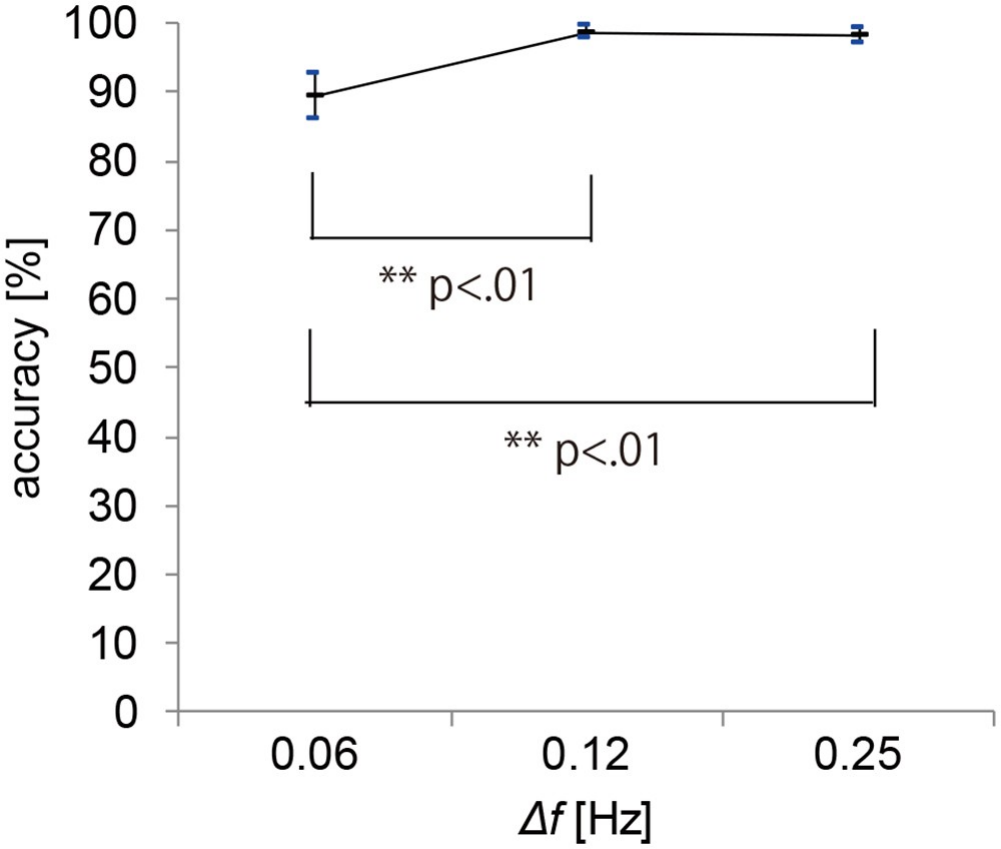
Experiment 2a: Average identification accuracy as a function of left-right frequency difference Δ*f*.

**Fig 8:**
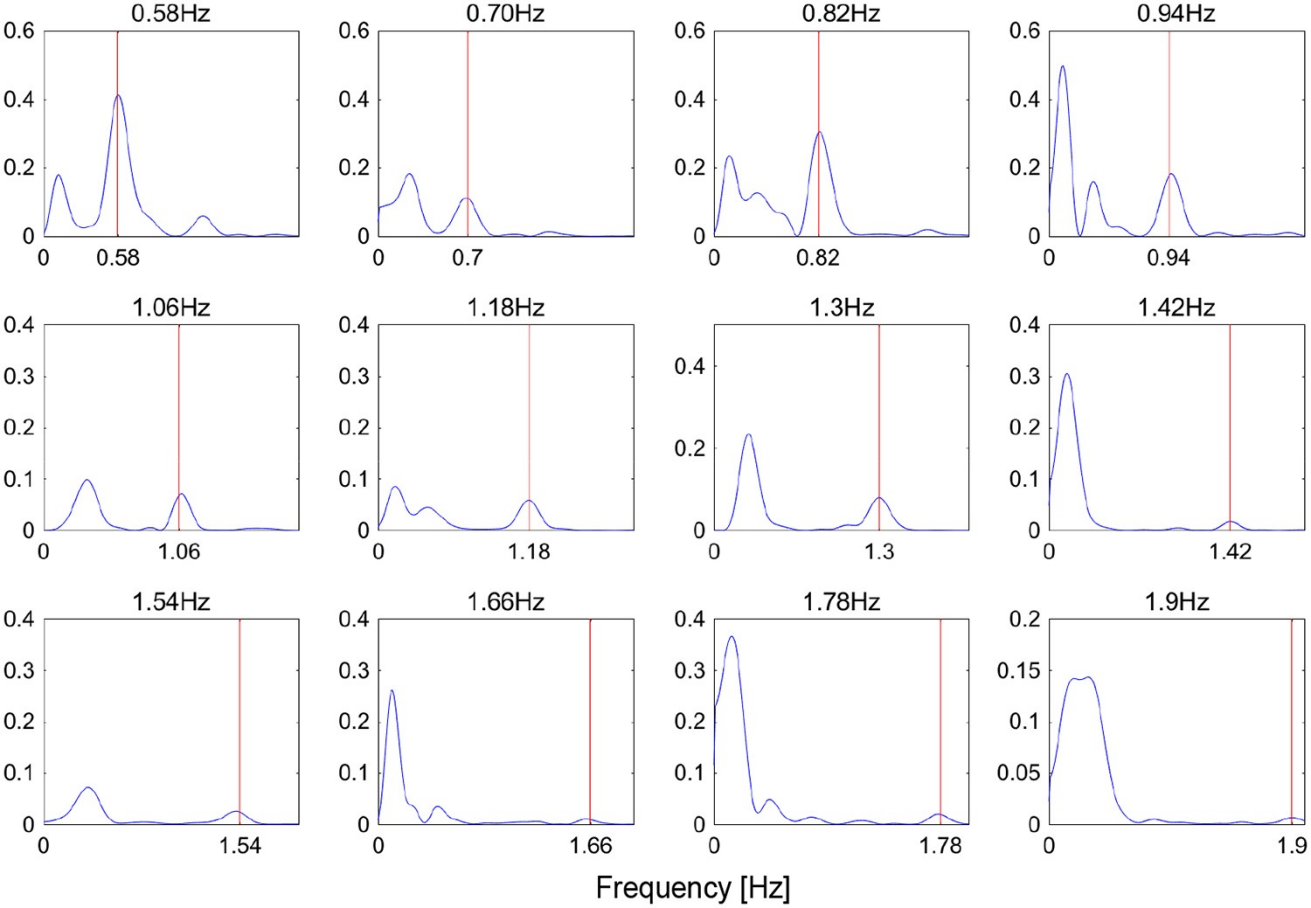
Experiment 2b: Typical PSD as a function of frequency for each frequency condition for a single participant.

**Fig 9:**
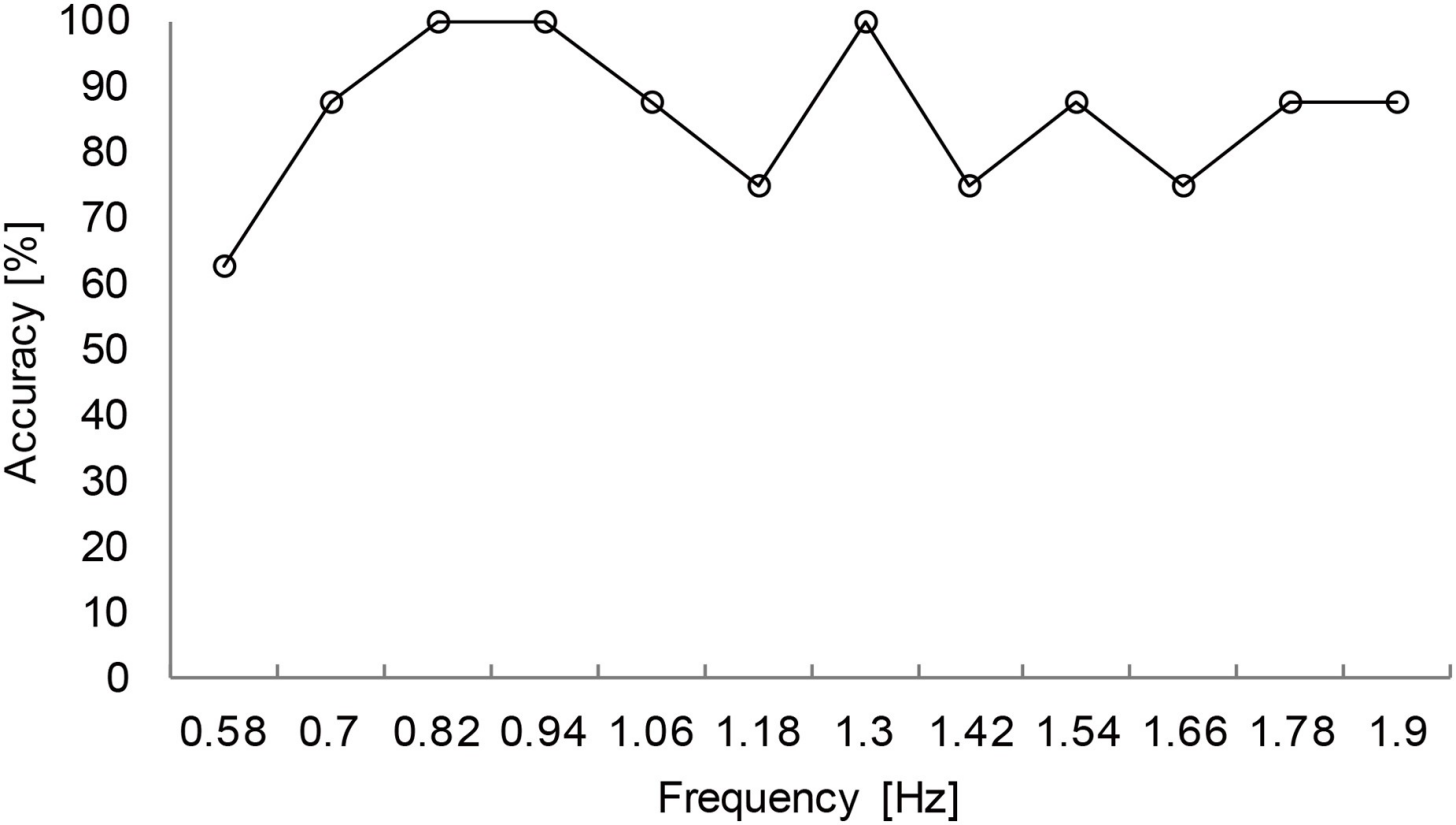
Experiment 2b: Identification accuracy averaged over all participants for the 12 luminance-modulation frequencies.

1. **Experiment 2a: Communication interface with two response options**
  The goal of experiment 2a was to identify at which circle the participants gazed based on pupil-size oscillation measured with a visual display. Fig 5 shows typical PSD results obtained with 0.01 Hz intervals. These results were constructed from the DFT of 7 s of time-series data of pupil response for one participant gazing at the right circle. The results show the PSD for differences in luminance-modulation frequencies between the two circles of Δ*f* = 0.06, 0.12, and 0.25 Hz. The blue and red lines indicate the PSD at the luminance-modulation frequency of the left and right circles, respectively. These results show that, in all of the trials, the PSD of the luminance-modulation frequency (red line) of the gaze-targeted circle (i.e., the right circle) exceeded that of the left circle (blue line). This indicates that, even when the difference between the luminance-modulation frequencies of the gaze-targeted stimulus and adjacent (i.e., non-gaze-targeted) stimuli is relatively small (0.06, 0.12, 0.25 Hz), it is possible to identify the gaze-targeted stimulus based on the pupil response.
  Fig 6 shows the accuracy with which the gaze-targeted visual stimulus is identified (called the “identification accuracy”) averaged over all participants for each frequency combination. The gaze-targeted stimulus is identified by comparing the PSD of the luminance-modulation frequencies of the two circles. Based on these results, we conclude that the gaze-targeted object can be identified with at least 85% accuracy except just one condition (Δ*f* = 0.06Hz, luminance-modulation frequency = 2Hz) tested in this experiment. Fig 7 shows the identification accuracy as a function of the frequency difference Δ*f* between the left and right circles for all base frequencies. The gaze-targeted stimulus was identified by comparing the PSD of the luminance-modulation frequencies of the two circles. In other words, we assume that, when a participant gazed at a circle with a certain luminance-modulation frequency, the PSD for the given frequency increased.
  For each frequency condition and for participants following the instructions regarding gaze position, we calculated the accuracy of the identification of the gaze-targeted stimulus based on the pupil response. The identification accuracy averaged over participants was 89.4%, 98.8%, and 98.1% for the frequency difference Δ*f* of 0.06, 0.12, and 0.25 Hz, respectively. We used a single-factor analysis of variance to investigate the quantitative effect of the frequency difference between gaze-targeted and non-gaze-targeted visual stimuli and found a significant effect [F (2,47) = 9.28, p < 0.01]. The results of multiple comparisons based on Fisher’s protected least significant difference method reveal that the differences in the results for Δ*f* = 0.06 Hz and Δ*f* = 0.12 Hz, and for Δ*f* = 0.06 Hz and Δ*f* = 0.25 Hz are significant at the level of 1%. Based on these analyses, a reliable estimate (98% accuracy) from the present method requires a minimum difference in luminance-modulation frequency between the two circles of 0.12 Hz. In experiment 2a, we identified the gaze-targeted circles (L circle or R circle) by comparing the frequency of the PSD peak of the pupil-size oscillation with the luminance-modulation frequencies of two circles without applying the correction (4). As shown in Fig 6, the identification accuracy is very high. In fact, recalculating the prediction with the correction (4) included does not change the results, which we attribute to the small difference Δ*f* in the luminance-modulation frequencies of the two circles. For greater differences Δ*f* in the luminance-modulation frequencies, the correction method should be included for a more reliable comparison between the two PSDs.
2. **Experiment 2b: Information-input interface with 12 options**
  Experiment 2b simultaneously compared 12 circles with different luminance-modulation frequencies and examined the identification accuracy of the gaze-targeted circle. Fig 8 shows typical PSDs calculated with 0.01 Hz, DFT sampling intervals for time-domain data spanning 7 s of pupil response. The results are given for each frequency condition for one participant. The red lines indicate the luminance-modulation frequency of the gaze-targeted circle. These results show that, in most of the cases, one of the PSD peaks corresponds to the luminance-modulation frequency of the gaze-targeted stimulus. In other words, even when simultaneously presenting 12 visual stimuli with different luminance-modulation frequencies, the pupil size synchronizes to the luminance-modulation frequency of the gaze-targeted stimulus.
  However, more often than not, the PSD peak at the luminance-modulation frequency of the gaze-targeted stimulus is a minor PSD peak. Therefore, we apply the data-correction method developed in experiment 1 to increase the identification accuracy. Specifically, the PSD functions shown in Fig 8 are multiplied by the weight *ω* given in Eq (4), and the frequency with the highest corrected PSD is taken as the frequency for identifying the gaze-targeted stimulus. Fig 9 shows the identification accuracy for each of the 12 luminance-modulation frequencies averaged over all participants, and Table 3 lists the accuracy averaged over all frequencies for each participant. These results indicate that information input is possible for all of participants tested with at least at 60% accuracy. Note that, upon excluding the results for 0.58 Hz because the identification accuracy is quite low (62.5%) at this frequency, the average accuracy reaches 87.5%.

**Table 3:** Experiment2b: Identification accuracy for each participant averaged over all luminance-modulation frequencies.

Participant No.	A	B	C	D	E	F	G	H	Average
Accuracy rate [%]	100	66.7	100	83.3	100	75	66.7	91.7	85.4

## Discussion

This study proposes an information-input interface that uses pupillometry and DFT analysis and focuses on the synchronization of pupil-size oscillation with luminance modulation of visual stimuli. We experiment with an interface with two response choices and an information-input interface with 12 response choices and evaluate the accuracy with which information can be input using the proposed method. Although pupil size is well known to oscillate in response to periodic luminance modulation of visual stimuli [see, e.g., [16] [17] [18] [19] [20]], no reports exist that detail the correspondence between the pupil-oscillation frequency and luminance-modulation frequency of stimuli in an information-input interface. In addition to the proposed information-input interface, we report several important findings regarding the pupil response to visual stimuli with various luminance-modulation frequencies.

In experiment 1, we examine the basic frequency characteristics of the pupil response by exposing the participants to a single circular object with a luminance-modulation frequency from 0.25 to 3.0 Hz. The analysis results are consistent with those from previous studies, which reported, for participants aged 20 to 30 years, synchronization between the frequency of oscillations in participant pupil size and the luminance-modulation frequency up to 3.15 Hz [see, e.g., [22]]. The PSD obtained from the DFT of the time-domain data depends on the frequency condition and, based on the results of a nonlinear regression analysis, can be approximated by an exponential function with a high degree of explanatory power. This result is attributed to the pupil-size modulation lessening with increasing luminance frequency. The PSD results were thus corrected for this effect in order to compare the PSD for stimuli with different luminance-modulation frequencies and select the frequency that produces the greatest PSD. Upon applying this correction method [see Eq (4)], the accuracy with which the gaze-targeted stimulus is identified based on its luminance-modulation frequency is 90% or more for frequencies from 0.75 to 2.75 Hz. Identification accuracy is 90% at 0.25 Hz and 80% at 0.5 Hz. At 3 Hz, the identification accuracy drops to 30%. From these considerations, we conclude that luminance-modulation frequencies from 0.75 to 2.25 Hz are optimal for a gaze-input system. The reason why the identification accuracy was low in the less than 0.5 Hz, would be that the change of pupil size is affected by the change in heart beat and respiration due to changes in the activation of the sympathetic nervous system that occur in the range of 0.15 to 0.40 Hz [25] [26] [27]. From 0.75 to 2.75 Hz, the visual stimulus may be identified with the proposed methods based on the gaze with an accuracy of 90%. Furthermore, we propose an algorithm to generate input information based on the data obtained.

In experiment 2a, we examined the effectiveness of an interface with two response choices. Participants gazed at one of the two visual stimuli in the display and the gaze-targeted stimulus was identified by comparing the measured frequency of pupil-size modulation with the luminance-modulation frequency of the gaze-targeted stimulus. The results demonstrate an identification accuracy of 89.4% for the difference Δ*f* = 0.06 Hz between the luminance-modulation frequencies of the stimuli, which rises to 98% or more for Δ*f* = 0.12 and 0.25 Hz. These results demonstrate the effectiveness of the proposed method and indicate that the pupil diameter becomes synchronized to the luminance modulation of the gaze-targeted stimulus rather than to that of adjacent, non-gaze-targeted stimuli. It was recently shown that the PLR is also affected by the luminance modulation of nearby, non-gaze-targeted stimuli [12] [28] [29]. Therefore, we consider that the proposed method is more efficient and reliable when participants direct both gaze and attention to a given visual stimulus.

Experiment 2b investigated the effectiveness of the proposed algorithm applied to an information-input interface containing 12 items presented in a keyboard configuration. Based on the results of experiment 1, the luminance of the visual stimuli was modulated at frequencies ranging from 0.58 to 1.9 Hz. The results show that the identification accuracy averaged over all participants was 85.4%, which indicates that the pupil-size oscillation becomes synchronized to the luminance-modulation frequency of the gaze-targeted stimulus (3°viewing angle), even if neighboring stimuli have differing luminance-modulation frequencies with a separation of 1°. However, the identification accuracy for the lowest luminance-modulation frequency of 0.58 Hz is only 62.5%, so we conclude that an appropriate range of luminance-modulation frequency for visual stimuli is from 0.7 to 1.9 Hz for a reliable and accurate information-input interface. Some further issues remain to be discussed to improve the proposed method. The first issue pertains to the individual differences observed in the accuracy with which the stimuli are identified. Although three participants had identification accuracies of 100% in experiment 2b, two participants had identification accuracies around 67%. One possible cause for this individual difference is a difference in experience with the interface. This issue is especially important when developing a human interface for handicapped people. We must develop an interface that all patients can use with ease without special experience or training. The second issue to consider is the interface design, which includes the stimulus configuration and the algorithm for choosing a gaze-targeted object. In experiment 2b, we arranged the 12 stimuli so that the luminance-modulation frequency increased from left to right and from top to bottom. However, this arrangement may not be optimal because the configuration of the stimuli and the procedure design should make the algorithm more accurate and allow the development of an easy-to-use interface. In addition, in this study, the luminance of the visual stimulus was modulated by a sine wave to reduce participant stress. However, a square-wave modulation may improve the accuracy because pupil size reportedly changes more under square-wave modulation of the luminance of the stimulus [18]. Finally, the third issue is how attention and gaze position affect the ability to direct the gaze at an object whose luminance is being modulated. As mentioned above, recent reports claim that the PLR is affected by the luminance of a visual stimulus in a neighboring position, even if the eye is not directed at that position [28] [29].

In the present study, the participants were instructed to direct both eye and attention to the visual stimulus, which helped to accurately identify the gaze-targeted object without being distracted by the luminance modulation of adjacent stimuli. However, if distracting objects are in the field of view, the accuracy with which gaze-targeted objects are identified might decrease. Conversely, the use of attention only without directing gaze toward an object may suffice as input. Such an interface would be helpful for locked-in patients. Therefore, further detailed investigations are warranted to look into how eye gaze and attention can be exploited to improve the information-input interface.

Changes in pupil size are known to depend on several factors. Psychological factors, such as alertness level [6], emotions [30] [31] [32], and mental workload [33] [34], affect the mediation of pupil size by the autonomic nervous system. Additionally, the mean steady-state pupil size reportedly decreases with age [35]. The impacts of these factors must be investigated to employ our proposed system in the future. This will require experiments involving many participants with different demographic characteristics, such as gender, age, and living environment.

Compared with other techniques for information-input interfaces that use biological signals, such as EEG, MEG, or optical topography, pupillometry has some important benefits. The proposed method for implementing an information-input interface using pupillometry puts a low burden on users, has minimal invasiveness, requires no training or experience, has high theoretical validity, and requires no calibration. However, online analysis will be required for real-life application of this method. In fact, we have begun performing online analysis. The results appear to be reliable [36], although we have not yet collected a sufficient amount of data. In the near future, we hope to develop a complete system that functions online.

## Conclusions

This study demonstrates an information-input interface that uses the synchronization of pupil oscillation with the luminance modulation of visual stimuli to identify the stimuli. The method identifies the gaze-targeted object from among many objects with differing luminance-modulation frequencies. The use of pupillometry for uncalibrated eye tracking in HCI has several possible practical applications. This method may be helpful for the design of interfaces that allow patients with severely limited motor function to communicate clearly with others.
